# A Pooled Analysis of 3 Phase II Trials of Salvage Nivolumab/Ipilimumab After Nivolumab in Renal Cell Carcinoma

**DOI:** 10.1093/oncolo/oyad298

**Published:** 2023-11-10

**Authors:** Rana R McKay, Katharina Leucht, Wanling Xie, Opeyemi Jegede, David A Braun, Michael B Atkins, Marc-Oliver Grimm, Toni K Choueiri

**Affiliations:** Department of Medicine, University of California San Diego, La Jolla, CA, USA; Department of Urology, University of California San Diego, La Jolla, CA, USA; Department of Urology, Universitaetsklinikum Jena, Jena, Germany; Department of Data Science, Dana-Farber Cancer Institute, Boston, MA, USA; Department of Data Science, Dana-Farber Cancer Institute, Boston, MA, USA; Department of Medicine, Yale University, New Haven, CT, USA; Department of Medicine, Georgetown University, Washington, DC, USA; Department of Urology, Universitaetsklinikum Jena, Jena, Germany; Department of Medicine, Dana-Farber Cancer Institute, Boston, MA, USA

**Keywords:** nivolumab, adaptive, ipilimumab, renal cell carcinoma, immunotherapy, response

## Abstract

**Background:**

Nivolumab plus ipilimumab has demonstrated improved survival for treatment-naïve advanced clear cell renal cell carcinoma (RCC). A series of clinical trials evaluated the effect of salvage nivolumab plus ipilimumab in patients without an objective response to nivolumab. Given the size and heterogeneity of these studies, we performed a pooled analysis to better inform the activity of nivolumab plus ipilimumab after nivolumab.

**Patients and Methods:**

Eligible patients included those with advanced clear cell RCC having received no prior immunotherapy. The primary objective was confirmed objective response rate (ORR) by investigator-assessment. Secondary objectives included progression-free survival (PFS) and overall survival (OS).

**Results:**

The analysis included 410 patients with clear cell RCC, of whom 340 (82.9%) had IMDC intermediate/poor risk disease, and 137 (33.4%) had prior treatment. The 16-18-week ORR to nivolumab prior to nivolumab plus ipilimumab was 22.7% (*n* = 93), and best ORR to nivolumab was 25.1% (*n* = 103). Two hundred and thirty (56.1%) patients treated with nivolumab received nivolumab plus ipilimumab at a median of 16 weeks (IQR 9-19) after initiation of nivolumab [27.0% (*n* = 62) with stable disease and 73.0% (*n* = 168) with progressive disease to nivolumab]. The ORR to nivolumab plus ipilimumab was 12.6% (*n* = 29). Six-month PFS on nivolumab plus ipilimumab was 37% (95% CI, 27-47). Median follow-up was 34.3 months and 3-year OS was 59% (95% CI, 53-64) from nivolumab start.

**Conclusion:**

A small subset of patients lacking a response to nivolumab derive benefit from salvage nivolumab plus ipilimumab. When possible, both drugs should be given in concomitantly, rather in an adaptive fashion.

## Teaser

A series of clinical trials evaluated the effect of salvage nivolumab plus ipilimumab in patients without an objective response to nivolumab. This article reports a pooled analysis to better inform the activity of nivolumab plus ipilimumab after nivolumab.

**Implications for Practice**

In this pooled analysis, the authors investigated nivolumab plus ipilimumab after nivolumab in clear cell renal cell carcinoma (RCC). Results showed that salvage nivolumab plus ipilimumab was frequently not feasible and resulted in limited efficacy. The authors highlight the activity of nivolumab monotherapy in patients naïve to prior immunotherapy in RCC. This analysis does not support the use of adaptive nivolumab plus ipilimumab in advanced clear cell RCC.

## Introduction

Immune checkpoint inhibitors are a critical component of the treatment of patients with advanced renal cell carcinoma (RCC). Nivolumab, a programmed death 1 (PD-1) inhibitor, was the first checkpoint inhibitor to enter the clinic for RCC, based on the results of CheckMate-025.^1^ In this study, nivolumab improved overall survival (OS) and objective response rate (ORR) compared to everolimus in patients with resistance to vascular endothelial growth factor receptor (VEGFR) tyrosine kinase inhibitor therapy. In addition, treatment was associated with lower grade 3 or higher toxicity, improved quality of life,^2^ and long-term efficacy^3^ compared to everolimus.

The combination of nivolumab with ipilimumab, a cytotoxic T-lymphocyte antigen 4 (CTLA-4) inhibitor, was tested in the frontline setting compared to sunitinib in the landmark CheckMate-214 study.^4^ In this trial, the combination of nivolumab and ipilimumab demonstrated improved ORR, progression-free survival (PFS), and OS in patients with International Metastatic Database Consortium (IMDC) intermediate and poor risk clear cell RCC.^4^ While immune-related adverse events were observed, therapy was associated with improved quality of life,^5^ treatment-free survival, and response duration, which was maintained out to a minimum follow up of 60 months.^6^

At the time that the combination of nivolumab plus ipilimumab entered into the clinic, there were questions as to whether all patients required dual checkpoint blockade for treatment intensification upfront or whether therapy could be adapted based on initial response to PD-1 blockade alone. In addition, studies investigating the efficacy and safety of nivolumab monotherapy in the treatment-naïve setting were lacking. Therefore, a series of independent adaptive phase II multi-institutional clinical trials (OMNIVORE NCT03203473; HCRN GU16-260 NCT03117309; TITAN-RCC NCT02917772) were designed to address these knowledge gaps and evaluate the activity of nivolumab plus ipilimumab in patients without an objective response to nivolumab (Supplementary Table S1). In the OMNIVORE trial (*n* = 83), 57 of 71 potentially eligible patients were allocated to escalated therapy with nivolumab and ipilimumab, and 2 patients developed a confirmed partial response (PR) (4%), and there were no complete responses (CRs).^7^ In HCRN GU16-260, 35 of 97 potentially eligible patients were allocated to escalated therapy, and the ORR was 11.4% with one CR^8,9^ In the TITAN-RCC trial, 139 of 207 patients received escalated therapy, with 19 (13.7%) experiencing a PR and 4 experiencing a CR after escalated therapy.^10,11^ Across these studies, there was significant heterogeneity in patient selection, intensity, dosing, and timing of escalated therapy with nivolumab plus ipilimumab. In addition, as these studies were launched, additional data from prospective trials and retrospective studies became available on the activity of immune-oncology (IO) therapy following progression on prior IO. Given the complexity of these studies and heterogeneity of the data on the adaptive and sequential activity of IO, we embarked on a pooled meta-analysis of these prospective adaptive IO studies in RCC.

## Methods

### Study Design and Summary of Studies

We conducted a pooled analysis of patients with advanced clear cell RCC enrolled on 3 multi-institutional, adaptive, open-label phase II clinical trials.^7,8,10,11^ Potentially eligible patients were recruited from medical oncology clinics. This study was conducted in accordance with International Conference on Harmonization guidelines for Good Clinical Practice and the principles of the Declaration of Helsinki. The study protocols were approved by the institutional review board for each participating institution. All patients provided written informed consent to the clinical trials in this analysis.

OMNIVORE enrolled patients with advanced RCC of any histology who were permitted to have received prior therapy excluding PD-1 pathway or CTLA-4 inhibitors.^7^ While the study enrolled patients of any histology, only patients with clear cell RCC were included in this analysis. Eligible patients were enrolled from October 2017 to July 2019 and received treatment as previously described.^7^ Patients received treatment with induction nivolumab and subsequent arm allocation was based on Response Evaluation Criteria in Solid Tumours (RECIST) version 1.1 criteria within 6 months of treatment initiation. Patients with a PR or CR within 6 months discontinued treatment with nivolumab and were observed, while patients with confirmed stable disease (SD) or progressive disease (PD) after a minimum of 2 but no more than 6 months received nivolumab plus ipilimumab (up to 2 doses) followed by maintenance nivolumab. Imaging was generally performed every 8 weeks. The primary endpoints were the proportion of patients with a maintained response at 1 year after nivolumab discontinuation (arm A) and proportion of patients with converted response after the addition of ipilimumab (arm B) based on investigator assessment. The target sample size was 83 patients as previously described.^7^

HCRN 16-260 enrolled patients with treatment naïve advanced RCC with clear cell RCC into cohort A and non-clear RCC into cohort B.^8,9^ Only cohort A was included in this analysis. Eligible patients were enrolled from May 2017 to December 2019 and received treatment as previously described.^8,9^ During part A of the study, patients received nivolumab monotherapy for up to 96 weeks. If patients experienced PD at any time or had a best response of SD at 48 weeks, they were potentially eligible for part B, wherein patients received treatment with nivolumab plus ipilimumab (up to 4 doses) followed by nivolumab monotherapy. Imaging was performed every 12 weeks, with one additional imaging done at 18 weeks. The primary endpoint of part A was 1-year landmark PFS on nivolumab, based on investigator assessment, accounting for tumor PD-L1 expression. The target sample size for cohort A was 120 patients as previously described.^8,9^

TITAN-RCC enrolled patients with advanced, International Metastatic Database Consortium (IMDC) intermediate or poor risk, RCC with a clear-cell component.^11^ Patients having received no prior systemic therapy were enrolled onto the first-line cohort and those having received one prior anti-angiogenic agent or temsirolimus were enrolled onto the second-line cohort. Eligible patient were enrolled from October 2016 to December 2018 and received treatment as previously described.^11^ All subjected initiated treatment with nivolumab monotherapy. Patients with early PD at week 8, deemed by the investigator to be clinically significant, or non-responders (SD or PD) at week 16 received 2-4 doses of nivolumab plus ipilimumab (initially 2 doses and if persistent SD or PD another 2 doses). Remaining patients continued nivolumab but could receive nivolumab plus ipilimumab for a later PD. The primary endpoint was investigator assessed ORR based on RECIST version 1.1. The target sample size was 207 patients has previously described.^11^

### Study Objectives

The primary objective of the pooled analysis was to investigate the ORR of nivolumab monotherapy at 16-18 weeks and best ORR to salvage nivolumab plus ipilimimab after nivolumab monotherapy. ORR, based on confirmed ORR assessment, was defined by investigator-assessed RECIST version 1.1 criteria as reported originally in the clinical trials. The ORR for patients receiving nivolumab plus ipilimumab boost used tumor measurements at the time of ipilimumab initiation as a new baseline. Secondary endpoints included PFS to nivolumab plus ipilimumab defined as time from salvage nivolumab plus ipilimumab initiation to radiographic progression or censored at time of last disease assessment date (available for OMNIVORE and HCRN 16-260 only) and OS defined from the start of nivolumab monotherapy at study entry to death from any cause or censored at last follow-up date. Adverse event data were previously reported and is not included in this analysis.^7,8,10,11^

### Statistical Analysis

Patient, disease, treatment, and outcomes data were collected for each study into a centralized database for central analysis. Patient demographic and disease characteristics at baseline were described using frequencies for categorical variables or median (interquartile range) for continuous values. ORR was compared between baseline groups with the Chi-square test or Fisher’s exact test (as appropriate). Distributions of PFS and OS were estimated using the method of Kaplan-Meier and comparison between baseline groups was performed by the log-rank test. The landmark approach was used to correlate ORR to nivolumab at 16-18 weeks with OS; hazard ratio was estimated from Cox regression adjusting for IMDC risk groups, previous treatment status, ECOG PS and age at study entry. Two-sided *P*-values were reported.

## Results

### Patient Characteristics

Overall, 410 patients with clear cell RCC were included in the analysis of whom 80 were from the OMNIVORE, 123 were from HCRN 16-260, and 207 were from TITAN-RCC. Baseline disease characteristics are detailed in Table 1. The majority of patients had not received any prior systemic therapy (66.6%). IMDC risk groups at the time of nivolumab monotherapy initiation included 17.1% of patients with favorable risk, 65.1% with intermediate risk, and 17.8% with poor risk disease. A total of 230 patients were included in the nivolumab plus ipilimumab response dataset of whom 57 were from OMNIVORE, 35 were from HCRN 16-260, and 138 patients were from TITAN-RCC (Supplementary Table S2, Supplementary Fig. S1).

**Table 1.** Patient demographic and disease characteristics at baseline.

	HCRN 16-260	OMNIVORE	Titan	Total
	(*N* = 123)	(*N* = 80)	(*N* = 207)	(*N* = 410)
	*N* (%)	*N* (%)	*N* (%)	*N* (%)
Age at study entry, median, IQR	65 (58-72)	61 (55-71)	65 (57-71)	64 (57-71)
Gender				
Female	34 (27.6)	14 (17.5)	60 (29.0)	108 (26.3)
Male	89 (72.4)	66 (82.5)	147 (71.0)	302 (73.7)
Race				
White	104 (84.6)	74 (92.5)	149 (72.0)	327 (79.8)
Black	11 (8.9)	1 (1.3)	1 (0.5)	13 (3.2)
Asian	4 (3.3)	0 (0)	1 (0.5)	5 (1.2)
Other	2 (1.6)	5 (6.3)	19 (9.2)	26 (6.3)
Unknown	2 (1.6)	0 (0)	37 (17.9)	39 (9.5)
Ethnicity				
Hispanic	7 (5.7)	3 (3.8)	0 (0)	10 (2.4)
Non-Hispanic	115 (93.5)	76 (95.0)	0 (0)	191 (46.6)
Unknown/NA	1 (0.8)	1 (1.3)	207 (100.0)	209 (51.0)
M-stage at diagnosis				
M0	24 (19.5)	21 (26.3)	0 (0)	45 (11.0)
M1	86 (69.9)	18 (22.5)	207 (100.0)	311 (75.9)
Mx/unknown	13 (10.6)	41 (51.3)	0 (0)	54 (13.2)
Sarcomatoid differentiation				
No	101 (82.1)	74 (92.5)	0 (0)	175 (42.7)
Yes	22 (17.9)	6 (7.5)	0 (0)	28 (6.8)
NA	0 (0)	0 (0)	207 (100.0)	207 (50.5)
ECOG performance status				
0	79 (64.2)	54 (67.5)	132 (63.8)	265 (64.6)
1	43 (35.0)	26 (32.5)	75 (36.2)	144 (35.1)
2	1 (0.8)	0 (0)	0 (0)	1 (0.2)
Received prior nephrectomy				
No	23 (18.7)	10 (12.5)	46 (22.2)	79 (19.3)
Yes	100 (81.3)	70 (87.5)	161 (77.8)	331 (80.7)
Lines of prior therapy				
0	123 (100.0)	41 (51.3)	109 (52.7)	273 (66.6)
≥1	0 (0)	39 (48.8)	98 (47.3)	137 (33.4)
Liver metastasis				
No	95 (77.2)	0 (0)	156 (75.4)	251 (61.2)
Yes	28 (22.8)	0 (0)	51 (24.6)	79 (19.3)
NA	0 (0)	80 (100.0)	0 (0)	80 (19.5)
IMDC risk groups				
Favorable	35 (28.5)	26 (32.5)	9 (4.3)	70 (17.1)
Intermediate	76 (61.8)	44 (55.0)	147 (71.0)	267 (65.1)
Poor	12 (9.8)	10 (12.5)	51 (24.6)	73 (17.8)
For salvage nivolumab plus ipilimumab dataset				
Number of patients included for meta-analysis^a^	35 (28.5)	57 (71.3)	138 (66.7)	230 (56.1)
ORR (to nivolumab) at ipilimumab initiation				
SD	0 (0)	23 (40.4)	39 (28.3)	62 (27.0)
PD	35 (100.0)	34 (59.6)	99 (71.7)	168 (73.0)
Weeks from first nivolumab dose to salvage ipilimumab initiation, median, IQR	23 (15-45)	14 (8-16)	16 (8-17)	16 (9-19)

^a^Please refer to Supplement Table S2 for patients inclusion/exclusion.

Abbreviations: NA: not available; IQR: interquartile range; ECOG: Eastern Cooperative Oncology Group; IMDC: International Metastatic Database Consortium; ORR: objective response rate; SD: stable disease; PD: progressive disease.

### Objective Response Rate to Nivolumab Monotherapy

The ORR to nivolumab monotherapy was 25.1% (95% CI, 21.0%-29.6%) overall [20.0% (*n* = 82) PR; 5.1% (*n* = 21) CR] and 22.7% (95% CI, 18.7%-27.1%) at week 16-18 [22.0% (*n* = 90) PR; 0.7% (*n* = 3) CR] (Table 2, Supplementary Table S3). In treatment naïve patients, the ORR to nivolumab monotherapy was 29.3% overall and 26.0% at week 16-18. In previously treated patients, the ORR to nivoluamb monotherapy was 16.8% overall and 16.1% at week 16-18. Subgroup analyses in the overall population demonstrated that patients who were treatment naïve compared to those having received prior treatment (26.0% vs. 16.1%, *P* = .023) and those with ECOG performance status of 0 compared to 1-2 (26.4% vs. 15.9%, *P* = .015) had improved ORR at 16-18 weeks.

**Table 2.** Overall and subgroup analysis of objective response rate to nivolumab monotherapy Response was defined as confirmed complete or partial response per RECIST 1.1.

	At 16-18 weeks (*N* = 410)	At 24 weeks (*N* = 203, HCRN 16-260 and OMNIVORE only)	During therapy (*N* = 410)
All subjects	93/410 (22.7%)	47/203 (23.2%)	103/410 (25.1%)
Prior treatment status			
Treatment naïve	71/273 (26.0%)	43/164 (26.2%)	80/273 (29.3%)
Previously treated	22/137 (16.1%)	4/39 (10.3%)	23/137 (16.8%)
IMDC risk groups			
Favorable	18/70 (25.7%)	19/61 (31.1%)	25/70 (35.7%)
Intermediate	59/267 (22.1%)	23/120 (19.2%)	62/267 (23.2%)
Poor	16/73 (21.9%)	5/22 (22.7%)	16/73 (21.9%)
Sarcomatoid differentiation			
No	36/175 (20.6%)	39/175 (22.3%)	44/175 (25.1%)
Yes	8/28 (28.6%)	8/28 (28.6%)	9/28 (32.1%)
NA	49/207 (23.7%)	0/0	50/207 (24.2%)
ECOG			
0	70/265 (26.4%)	36/133 (27.1%)	77/265 (29.1%)
1	23/145 (15.9%)	11/70 (15.7%)	26/145 (17.9%)

Abbreviation: NA: not available.

### Objective Response Rate to Nivolumab Plus Ipilimumab

A total of 230 patients received nivolumab plus ipilimumab of whom 73.0% had PD and 27.0% had SD to nivolumab monotherapy at the time of salvage nivolumab plus ipilimumab initiation. Salvage nivolumab plus ipilimumab was initiated at a median of 16 weeks (interquartile range 9-19) from nivolumab initiation. The ORR to nivolumab plus ipilimumab was 12.6% (95% CI, 8.6%-17.6%) in the 230 patients receiving salvage nivolumab plus ipilimumab [10.4% (*n* = 24) PR; 2.2% (*n* = 5) CR]. In treatment naïve patients, the ORR was 11.8% [10.2% (*n* = 13) PR; 1.6% (*n* = 2) CR] and in previously treated patients the ORR was 13.6% [10.7% (*n* = 11) PR; 2.9% (*n* = 3) CR] (Table 3, Supplementary Tables S4). Subgroup analyses by prior treatment status, IMDC risk group, response to nivolumab, and timing of initiation of ipilimumab did not identify subgroups with statistically significant differential responses to salvage to nivolumab and ipilimumab (Table 3).

**Table 3.** Overall and subgroup analysis of best objective response to salvage nivolumab plus ipilimumab (*n* = 230). Objective response rate assessment used tumor measurements at salvage nivolumab plus ipilimumab initiation as baseline.

	No. of patients	No. (%) of confirmed CR/PR	*P*-value
All subjects	230	29 (12.6%)	
Prior treatment status			
Treatment naïve	127	015 (11.8%)	.69
Previously treated	103	014 (13.6%)	
IMDC risk groups			
Favorable	35	005 (14.3%)	.37
Intermediate	158	022 (13.9%)	
Poor	37	002 (5.4%)	
Response to nivolumab at ipilimumab start			
SD	62	005 (8.1%)	.21
PD	168	024 (14.3%)	
Best ORR to nivolumab monotherapy			
CR	3	002 (66.7%)	NR
PR	21	004 (19.0%)	
SD	86	007 (8.1%)	
PD	120	016 (13.3%)	
Best ORR to nivolumab monotherapy			
Response (confirmed CR/PR)	24	006 (25.0%)	.09
Non-response	206	023 (11.2%)	
Initiation of ipilimumab within 18 weeks			
No	57	008 (14.0%)	.71
Yes	173	021 (12.1%)	
Initiation of ipilimumab within 24 weeks			
No	42	006 (14.3%)	.72
Yes	188	023 (12.2%)	

Abbreviations: CR: complete response; PR: partial response; IMDC: International Metastatic RCC Database Consortium; SD: stable disease; PD: progressive disease; ORR: objective response rate; NR: not reported.

### Progression-Free Survival After Salvage Nivolumab Plus Ipilimumab Initiation

**Figure 1.**
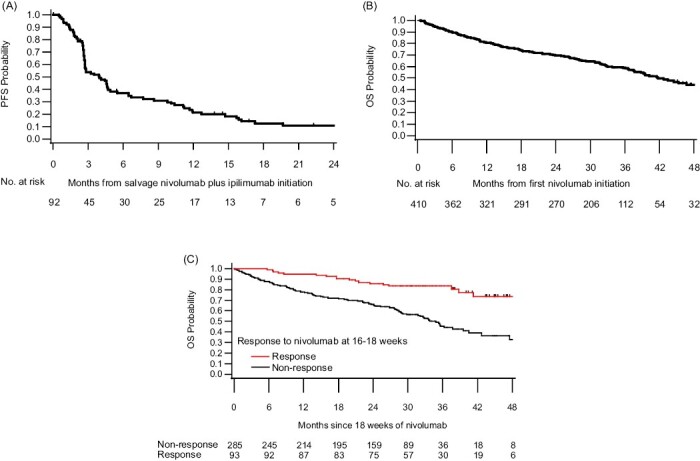
Kaplan-Meier curve for (**A**) progression-free survival from salvage ipilimumab initiation (available only for OMNIVORE and HCRN 16-260, *n* = 92), (**B**) overall survival from nivolumab initiation (overall cohort, *n* = 410), and (**C**) overall survival according to objective response to nivolumab at 16-18 weeks (landmark time, *N* = 378^a^). (A) Excluding 32 patients who died or withdrew before the landmark time.

The median PFS after initiation of salvage nivolumab plus ipilimumab was 3.9 (95% CI, 2.7-4.7) months and 6-month PFS was 37% (95% CI, 27-47) (for OMNIVORE and HCRN 16-260 only, *n* = 92) (Fig. 1A). Subgroup analysis demonstrated that favorable IMDC risk group (6-month PFS 40% vs. 37% vs. 29% in favorable, intermediate, and poor risk disease respectively, *P* = .048) and having SD (vs. PD) as ORR to nivolumab at ipilimumab initiation (6-month PFS 68% vs. 27%, *P* = .008) were associated with longer PFS to salvage nivolumab plus ipilimumab (Table 4, Supplementary Fig. S2).

**Table 4.** Overall and subgroup analysis of progression-free survival after initiation of ipilimumab. Available only for OMNIVORE and HCRN 16-260 (*n* = 92).

	No. of patients	No. of events	6 months PFS % (95% CI)	Log-rank *P*-value
All patients	92	77	37 (27-47)	
Prior treatment status				
Treatment naïve	60	49	38 (25-51)	.56
Previously treated	32	28	35 (19-52)	
IMDC risk groups				
Favorable	28	20	40 (20-59)	.048
Intermediate	57	50	37 (24-49)	
Poor	7	7	29 (4-61)	
Objective response to nivolumab at ipilimumab start				
SD	23	17	68 (44-83)	.008
PD	69	60	27 (17-38)	
Initiation of ipilimumab within 18 weeks				
No	29	26	32 (16-49)	.96
Yes	63	51	40 (27-52)	
Initiation of ipilimumab within 24 weeks				
No	20	18	32 (13-52)	.95
Yes	72	59	39 (27-50)	
Timing of ipilimimab start and objective response (to nivolumab) at ipilimumab start				
>24weeks, PD	20	18	32 (13-52)	NR
<24weeks, SD	23	17	68 (44-83)	
<24weeks, PD	49	42	25 (13-38)	
Combination of best ORR and ORR at ipilimumab start				
SD only	23	17	68 (44-83)	NR
SD to PD	14	11	33 (10-59)	
CR/PR to PD	8	8	25 (4-56)	
PD only	47	41	25 (14-39)	

Abbreviations: PFS: progression-free survival; CI: confidence interval; IMDC: International Metastatic RCC Database Consortium; SD: stable disease; PD: progressive disease; CR: complete response; PR: partial response; NR: not reported.

### Overall Survival from Nivolumab Initiation

Overall, there have been 177 deaths and median follow up for alive patients was 34.3 months (range 32.2-36.8 months across the trials) (Supplementary Table S5). The 3-year OS from nivolumab initiation was 59% (95% CI, 53-64) (Fig. 1B). Subgroup analysis demonstrated that IMDC risk group was associated with OS (3-year OS 86% vs. 59% vs. 29% for favorable, intermediate, and poor risk disease, respectively, *P* < .0001) (Supplementary Table S6). Furthermore, in a landmark analysis, achieving an objective response to nivolumab at 16-18 weeks was associated with prolonged OS compared to non-responders (adjusted hazard ratio = 0.28, 95% CI, 0.17-0.46, *P* < .0001; Fig. 1).

## Discussion

In this pooled analysis of 3 multi-institutional phase II studies, we investigate the activity of nivolumab monotherapy and salvage nivolumab and ipilimumab in patients with advanced RCC. To our knowledge, this is the largest series to date investigating an adaptive IO treatment strategy in patients with advanced RCC. In this analysis, we identify several novel insights into the activity of nivolumab and salvage nivolumab and ipilimumab. Overall, our findings do not support an adaptive IO treatment strategy.

First, we confirm that nivolumab has activity as a single agent in patients with advanced clear cell RCC. Given that our analysis primarily evaluated the activity of nivolumab by week 16-18, we likely underestimate the ORR observed in prior studies. In our study, the ORR at week 16-18 in patients with clear cell RCC was 22.7% overall, 26.0% in treatment naïve individuals, and 16.1% in previously treated individuals. Furthermore, the OMNIVORE and TITAN-RCC study initiated salvage nivolumab plus ipilimumab as early as 8 weeks to up to 24 weeks from nivolumab monotherapy initiation and the HCRN 16-260 initiated salvage nivolumab plus ipilimumab at PD or 48 weeks for patients with SD, further supporting that the nivolumab monotherapy ORR is likely an underestimation in our analysis. As a point of reference, the ORR from the CheckMate-025 study of nivolumab in the refractory setting was 22.9% with 1.0% of patients experiencing a CR.^1,3^ Additionally, we demonstrate lower ORR and CRs than that observed with frontline nivolumab plus ipilimumab in the CheckMate-214 study.^4^ At the present time, further prospective data on the activity of nivolumab in the frontline setting are lacking. The phase III CheckMate-8Y8 (NCT03873402) comparing nivolumab plus ipilimumab to nivolumab plus placebo with coprimary endpoint of ORR and PFS by independent review will provide further insights on the contribution of ipilimumab to nivolumab in treatment naïve patients with intermediate or poor-risk clear cell RCC. The KEYNOTE-427 study (cohort A), a single arm phase II study, investigated the activity of pembrolizumab in patients with treatment naïve clear cell RCC.^12^ In this study, pembrolizumab demonstrated an ORR of 36.4% with 3.6% CR.^12^ The median time to response was 2.8 months with range of 2.5-12.9 months, highlighting that delayed responses can be observed with IO therapy.^12^

Second, we demonstrate that salvage nivolumab plus ipilimumab has limited efficacy with low rates of response conversion in patients with SD or PD to nivolumab. Additionally, CRs were limited. In our study, 73.9% of patients had PD at time of ipilimumab initiation. These patients either had primary resistance or early acquired resistance suggesting a biologically distinct subgroup of patients being challenged with ipilimumab. Since these studies were launched, additional data from prospective trials became available on the activity of IO therapy following progression on prior IO. The FRACTION-RCC tested the activity of nivolumab plus ipilimumab combination in patients who progressed during or after IO therapy and demonstrated an ORR of 17.4% with no CRs.^13^ KEYNOTE-146 evaluated pembrolizumab plus lenvatinib in IO-pretreated individuals and demonstrated an ORR of 62.5% with no observed CRs.^14^ More recently, CONTACT-3, the first reported randomized phase III trial evaluating IO post IO, tested atezolizumab plus cabozantinib versus cabozantinib in patients having progressed during or after IO.^15^ The study demonstrated no difference in PFS, OS, or ORR with the combination compared to cabozantinib. In aggregate, these data highlight the limited role for IO following disease progression on prior IO therapy.

Third, we demonstrate that a substantial number of patients were unable to receive ipilimumab with this adaptive strategy. Of the 307 patients without a response to nivolumab monotherapy, 33% never received salvage nivolumab plus ipilimumab, highlighting that upfront treatment intensification is preferred to maximize outcomes for patients. Additionally, of the 103 (25%) with a response to nivolumab, 23% ultimately developed disease progression warranting nivolumab plus ipilimumab initiation. In light of these data, there is limited role for salvage nivolumab plus ipilimumab given inability to salvage non-responders and lack of durability associated with this strategy.

The tumor immune microenvironment plays an important role in RCC response to immunotherapy. Our data suggests that an adaptive strategy that does not include upfront treatment intensification results in suboptimal responses. Single-cell RNA-seq analysis across clear cell RCC disease states revealed that terminally exhausted CD8+ T cells and M2-like tumor associated macrophages were enriched in advanced RCC and associated with disease progression to nivolumab.^16,17^ Thus, there is a need to maximally target these resistant cells upfront to prevent disease progression and optimize the immune response. While existing IO treatments have focused on improving T-cell function through inhibition of PD-1 or CTLA-4, multiple other T-cell inhibitory pathways and the myeloid compartment play a significant role in the immune dysfunction in RCC and combination IO strategies that target multiple components of the TME are likely warranted.

Although this was a pooled analysis of 3 prospective clinical trials, there are several limitations. Each of these trials had specific enrollment criteria, procedures for ipilimumab escalation, timing of ipilimumab, and dosing of ipilimumab. To ensure homogeneity in the analysis, we largely reported on outcomes that were uniform across all 3 studies. Additionally, the studies enrolled patients during the same time period and follow-up times for each of the 3 studies were similar.

In summary, this pooled analysis addresses important clinically relevant questions in the field regarding salvage IO strategies in patients with RCC. In line with other studies, this work establishes the activity of nivolumab monotherapy in patients with advanced RCC. Using a response-adaptive design, these studies demonstrate the limited activity of nivolumab plus ipilimumab salvage in patients lacking a response to nivolumab, and this approach has limited application in clinical practice.

## Supplementary Material

Supplementary material is available at *The Oncologist* online.

oyad298_suppl_Supplementary_Tables_S1-S6

oyad298_suppl_Supplementary_Figures_S1-S2

## Data Availability

The data underlying this article will be shared on reasonable request to the corresponding author.
