# Supplementary material for: A Pooled Analysis of 3 Phase II Trials of Salvage Nivolumab/Ipilimumab After Nivolumab in Renal Cell Carcinoma
Source: Oncologist. 2023 Nov 10;29(4):324–31. doi: 10.1093/oncolo/oyad298 (PMC10994246; doi:10.1093/oncolo/oyad298)
Supplement: oyad298_suppl_Supplementary_Tables_S1-S6 [file oyad298_suppl_supplementary_tables_s1-s6.docx]

**Supplemental Material:**

**Supplement Table S1.** Summary of key design elements of OMNIVORE, HCRN GU16-260, and TITAN-RCC trials.

|  | **OMNIVORE** | **HCRN GU16-260** | **TITAN-RCC** |
| --- | --- | --- | --- |
| Prior systemic therapy permitted | Yes | No | Yes |
| Non-clear cell RCC permitted | Yes | Yes | No |
| Doses of ipilimumab permitted | 2 | 4 | 2 and if persistent SD/PD additional 2 |
| Timing of ipilimumab initiation | 16-24 weeks (if SD/PD, Arm B) or later PD (if CR/PR, Arm A) | At PD or 48-week SD | 8-16 weeks (if SD/PD) or later PD (if CR/PR) |
| Primary endpoint | 1-year maintained response (Arm A); Converted ORR after ipilimumab addition (Arm B) | 1-year PFS on nivolumab | ORR at any time after nivolumab initiation |

RCC=Renal cell carcinoma; SD=Stable disease; PD=Progressive disease; ORR=Objective response rate; PFS=Progressive-free survival.

**Supplement Table S2.** Number of subjects included for the meta-analysis.

|  | **Part 1 – Nivolumab** | | | **Part 2 -Salvage Nivolumab** **plus ipilimumab** | | |
| --- | --- | --- | --- | --- | --- | --- |
|  | **Total** | **Clear cell** | **Non-clear cell** | **Total** | **Clear cell** | **Non-clear cell** |
| HCRN 16-260 | 158 | 123 | 35 | 52 | 35 | 17 |
| OMNIVORE | 83 | 80 | 3 | 59 ^A^ | 57 | 2 |
| Titan | 207 | 207 | 0 | 138^B^ | 138 | 0 |
| Total | 448 | 410 | 38 | 249 | 230 | 19 |
| Number of subjects included for meta-analysis |  | 410 |  |  | 230 |  |

1. OMNIVORE: N=59, including 56 subjects from Arm-B (who received ipilimumab within 6 months due to no response to nivolumab) and 3 subjects from Arm-A (who initially responded to nivolumab but subsequently progressed).
2. Titan: 139 patients got at least one dose of ipilimumab. One subject was excluded because he received an additional cancer treatment in parallel to the study prior to the initiation of ipilimumab.

**Supplement Table S3.** Summary of objective response to therapies in clear cell histology across clinical trials.

|  | **Trial** | | |  |
| --- | --- | --- | --- | --- |
|  | **HCRN GU16-260** | **OMNIVORE** | **TITAN-RCC** | **Total** |
| **Best ORR to nivolumab at 16-18 weeks** | | | | |
|  | **(N=123)** | **(N=80)** | **(N=207)** | **(N=410)** |
| CR | 001 (0.8%) | 000 (0.0%) | 002 (1.0%) | 003 (0.7%) |
| PR | 034 (27.6%) | 009 (11.3%) | 047 (22.7%) | 090 (22.0%) |
| uPR | 002 (1.6%) | 002 (2.5%) | 000 (0.0%) | 004 (1.0%) |
| SD | 049 (39.8%) | 035 (43.8%) | 052 (25.1%) | 136 (33.2%) |
| PD | 031 (25.2%) | 034 (42.5%) | 099 (47.8%) | 164 (40.0%) |
| NE | 006 (4.9%) | 000 (0.0%) | 007 (3.4%) | 013 (3.2%) |
|  |  |  |  |  |
| **Best ORR to nivolumab at 24 weeks (HCRN and OMNIVORE only)** | | | | |
|  | **(N=123)** | **(N=80)** | **(N=0)** | **(N=203)** |
| CR | 003 (2.4%) | 000 (0.0%) | NA | 003 (1.5%) |
| PR | 035 (28.5%) | 009 (11.3%) | NA | 044 (21.7%) |
| uPR | 002 (1.6%) | 002 (2.5%) | NA | 004 (2.0%) |
| SD | 046 (37.4%) | 035 (43.8%) | NA | 081 (39.9%) |
| PD | 032 (26.0%) | 034 (42.5%) | NA | 066 (32.5%) |
| NE | 005 (4.1%) | 000 (0.0%) | NA | 005 (2.5%) |
|  |  |  |  |  |
| **Best ORR to nivolumab monotherapy** | | | | |
|  | **(N=123)** | **(N=80)** | **(N=207)** | **(N=410)** |
| CR | 010 (8.1%) | 001 (1.3%) | 010 (4.8%) | 021 (5.1%) |
| PR | 033 (26.8%) | 009 (11.3%) | 040 (19.3%) | 082 (20.0%) |
| uPR | 000 (0.0%) | 002 (2.5%) | 000 (0.0%) | 002 (0.5%) |
| SD | 041 (33.3%) | 034 (42.5%) | 052 (25.1%) | 127 (31.0%) |
| PD | 034 (27.6%) | 034 (42.5%) | 101 (48.8%) | 169 (41.2%) |
| NE | 005 (4.1%) | 000 (0.0%) | 004 (1.9%) | 009 (2.2%) |
|  |  |  |  |  |
| **Best ORR to salvage nivolumab plus ipilimumab** | | | | |
|  | \| **(N=35)** \| \| --- \| | **(N=57)** | **(N=138)** | **(N=230)** |
| CR | 001 (2.9%) | 000 (0.0%) | 004 (2.9%) | 005 (2.2%) |
| PR | 003 (8.6%) | 002 (3.5%) | 019 (13.8%) | 024 (10.4%) |
| SD | 009 (25.7%) | 026 (45.6%) | 055 (39.9%) | 090 (39.1%) |
| PD | 017 (48.6%) | 024 (42.1%) | 051 (37.0%) | 092 (40.0%) |
| NE | 005 (14.3%) | 005 (8.8%) | 009 (6.5%) | 019 (8.3%) |

ORR=Objective response rate; CR=Complete response; PR=Partial response; uPR=Unconfirmed partial response; SD=Stable disease; PD=Progressive disease; NE=Not evaluable.

**Supplement Table S4.** Summary of objective response rates to therapies according to previous treatment status.

|  | **Treatment naïve** | **Previously Treated** | **Total** |
| --- | --- | --- | --- |
| **Best ORR to nivolumab at 16-18 weeks** | **(N = 273)** | **(N = 137)** | **(N = 410)** |
| CR | 3 (1.1%) | 0 (0%) | 3 (0.7%) |
| PR | 68 (24.9%) | 22 (16.1%) | 90 (22.0%) |
| uPR | 4 (1.5%) | 0 (0%) | 4 (1.0%) |
| SD | 102 (37.4%) | 34 (24.8%) | 136 (33.2%) |
| PD | 87 (31.9%) | 77 (56.2%) | 164 (40.0%) |
| NE | 9 (3.3%) | 4 (2.9%) | 13 (3.2%) |
| **Best ORR to nivolumab at 24 weeks (HCRN 16-260 and OMNIVORE only)** | **(N=164)** | **(N=39)** | **(N=203)** |
| CR | 3 (1.8%) | 0 (0%) | 3 (1.5%) |
| PR | 40 (24.4%) | 4 (10.3%) | 44 (21.7%) |
| uPR | 4 (2.4%) | 0 (0%) | 4 (2.0%) |
| SD | 70 (42.7%) | 11 (28.2%) | 81 (39.9%) |
| PD | 42 (25.6%) | 24 (61.5%) | 66 (32.5%) |
| NE | 5 (3.0%) | 0 (0%) | 5 (2.5%) |
| **Best ORR to nivolumab monotherapy** | **(N = 273)** | **(N = 137)** | **(N = 410)** |
| CR | 18 (6.6%) | 3 (2.2%) | 21 (5.1%) |
| PR | 62 (22.7%) | 20 (14.6%) | 82 (20.0%) |
| uPR | 2 (0.7%) | 0 (0%) | 2 (0.5%) |
| SD | 94 (34.4%) | 33 (24.1%) | 127 (31.0%) |
| PD | 90 (33.0%) | 79 (57.7%) | 169 (41.2%) |
| NE | 7 (2.6%) | 2 (1.5%) | 9 (2.2%) |
| **Best ORR to salvage nivolumab plus ipilimumab** | **(N=127)** | **(N=103)** | **(N=230)** |
| CR | 2 (1.6%) | 3 (2.9%) | 5 (2.2%) |
| PR | 13 (10.2%) | 11 (10.7%) | 24 (10.4%) |
| SD | 51 (40.2%) | 39 (37.9%) | 90 (39.1%) |
| PD | 50 (39.4%) | 42 (40.8%) | 92 (40.0%) |
| NE | 11 (8.7%) | 8 (7.8%) | 19 (8.3%) |

ORR=Objective response rate; CR=Complete response; PR=Partial response; uPR=Unconfirmed partial response; SD=Stable disease; PD=Progressive disease; NE=Not evaluable.

**Supplement Table S5.** Summary of data cutoff and median follow-up across clinical trials.

| **Trial** | **Total** | **Number of deaths** | **Number alive** | **Median follow-up**  **in alive patients (months)** | **Data cutoff date** |
| --- | --- | --- | --- | --- | --- |
| HCRN 16-260 | 123 | 47 | 76 | 36.8 | August 2022 |
| OMNIVORE | 80 | 28 | 52 | 32.2 | December 2022 |
| TITAN-RCC | 207 | 102 | 105 | 34.0 | October 2021 |
| **Total** | 410 | 177 | 233 | 34.3 |  |

**Supplement Table S6.** Summary of overall survival from nivolumab initiation.

|  | **Number**  **of patients** | **Number**  **of events** | **3-years OS, %**  **(95% CI)** | **Log-rank**  **P-value** |
| --- | --- | --- | --- | --- |
| **All patients** | 410 | 177 | 59 (53-64) |  |
| **Prior treatment status** |  |  |  |  |
| Treatment naïve | 273 | 111 | 62 (56-68) | 0.06 |
| Previously Treated | 137 | 66 | 52 (43-61) |  |
| **IMDC risk groups** |  |  |  |  |
| Favorable | 70 | 16 | 86 (74-93) | <0.0001 |
| Intermediate | 267 | 116 | 59 (52-65) |  |
| Poor | 73 | 45 | 29 (18-42) |  |
| **Sarcotomaid histology^A^** |  |  |  |  |
| No | 175 | 61 | 69 (61-76) | 0.12 |
| Yes | 28 | 14 | 54 (34-71) |  |

OS=Overall survival; CI=Confidence interval; IMDC=International Metastatic RCC Database Consortium.

1. Excluded TITAN-RCC that did not collect data on sarcotomaid histology.
