# Supplementary material for: A Pooled Analysis of 3 Phase II Trials of Salvage Nivolumab/Ipilimumab After Nivolumab in Renal Cell Carcinoma
Source: Oncologist. 2023 Nov 10;29(4):324–31. doi: 10.1093/oncolo/oyad298 (PMC10994246; doi:10.1093/oncolo/oyad298)
Supplement: oyad298_suppl_Supplementary_Figures_S1-S2 [file oyad298_suppl_supplementary_figures_s1-s2.docx]

**Supplement Figure S1.** Consort diagram of included trials. *Including 54 subjects from Arm-B (who received ipilimumab within 6 months due to no response to nivolumab) and 3 subjects from Arm-A (who initially responded to nivolumab but subsequently progressed). ccRCC=Clear cell renal cell carcinoma; nccRCC=Non-clear cell renal cell carcinoma; NP=Non-protocol therapy.


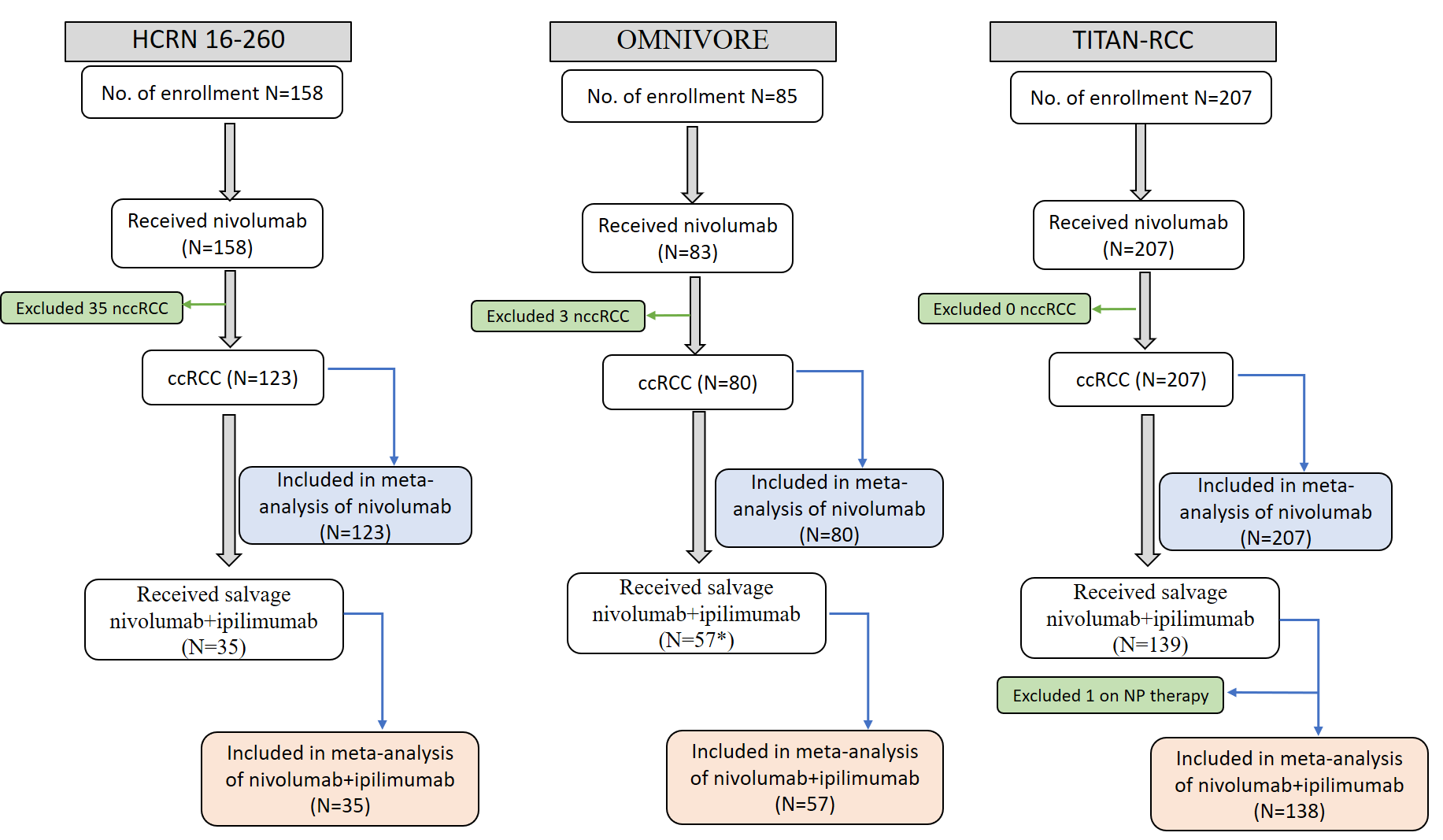


**Supplemental Figure S2.** Kaplan Meier estimates of progression-free survival (PFS) from salvage ipilimumab initiation, stratified by (A) IMDC risk group (favorable, intermediate, vs. poor) and (B) objective response to nivolumab at ipilimumab initiation [stable disease (SD) vs. progressive disease (PD)].

(A)

**
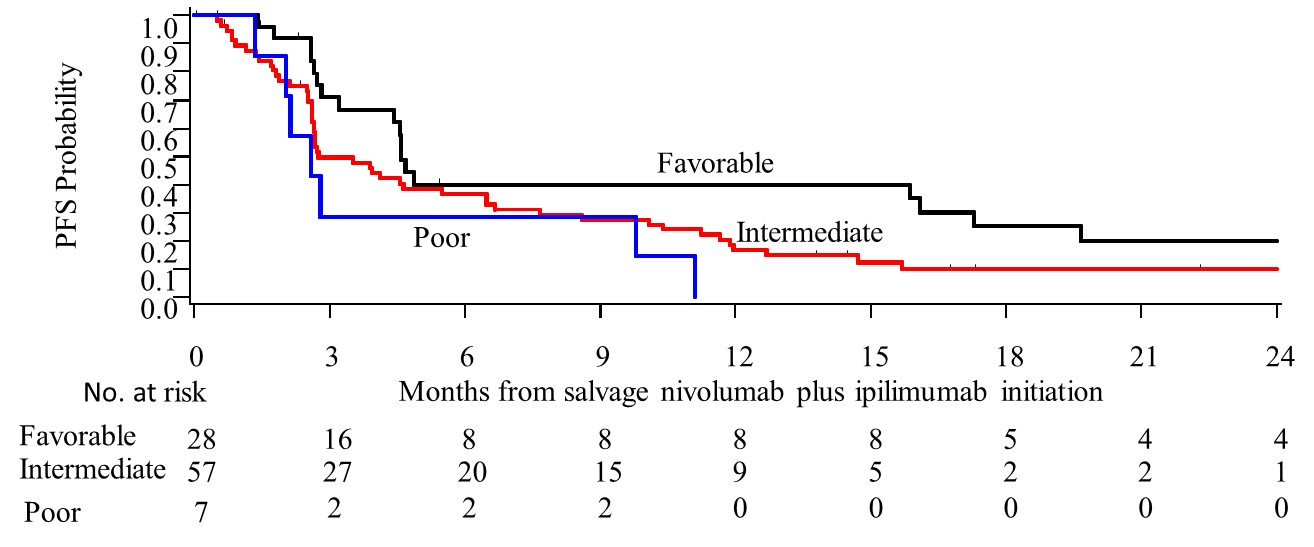
**

(B)

**
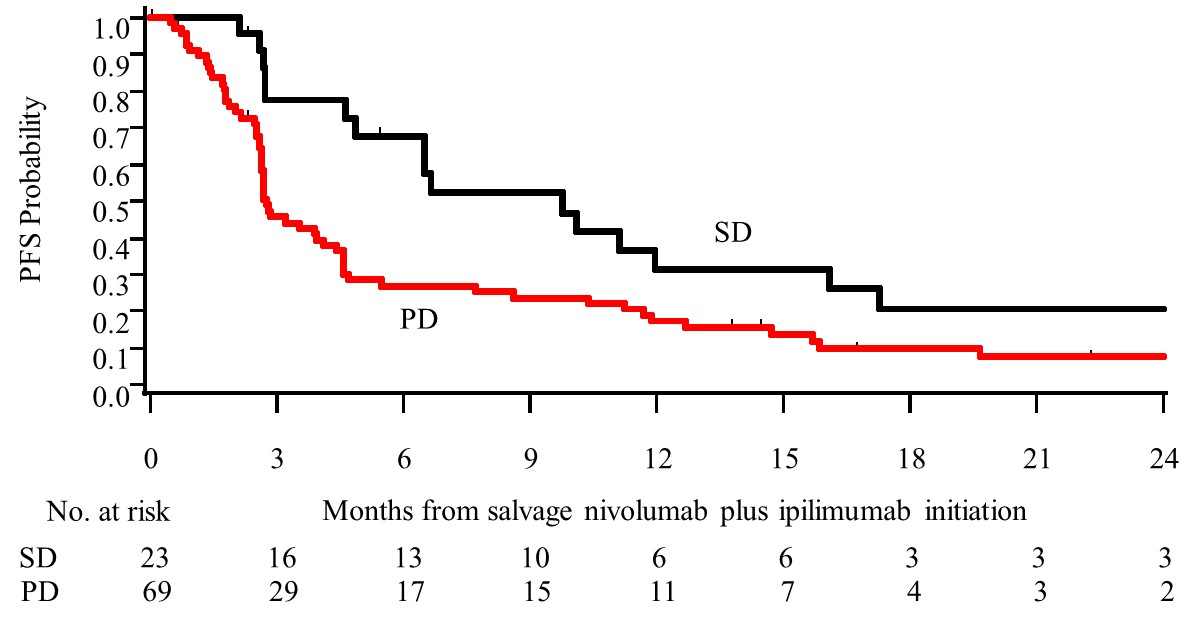
**
